# Proteogenomic characterization and comprehensive integrative genomic analysis of human colorectal cancer liver metastasis

**DOI:** 10.1186/s12943-018-0890-1

**Published:** 2018-09-21

**Authors:** Yu-Shui Ma, Tao Huang, Xiao-Ming Zhong, Hong-Wei Zhang, Xian-Ling Cong, Hong Xu, Gai-Xia Lu, Fei Yu, Shao-Bo Xue, Zhong-Wei Lv, Da Fu

**Affiliations:** 1 Central Laboratory for Medical Research, Shanghai Tenth People’s Hospital, Tongji University School of Medicine, Middle 301 Yanchang Road, Shanghai, 200072 China; 2 Shanghai Engineering Research Center of Molecular Therapeutics and New Drug Development, College of Chemistry and Molecular Engineering, East China Normal University, Shanghai, 200062 China; 3 Department of Nuclear Medicine, Shanghai Tenth People’s Hospital, Tongji University School of Medicine, Shanghai, 200072 China; 4 Institute of Health Sciences, Shanghai Institutes for Biological Sciences, Chinese Academy of Sciences, Shanghai, 200025 China; 5 Department of Radiology, Jiangxi Provincial Tumor Hospital, Nanchang, 330029 China; 6 Analytical Chemistry Platforms, Shanghai Institute for Advanced Immunochemical Studies, ShanghaiTech University, Shanghai, 201210 China; 7 Tissue Bank, China-Japan Union Hospital, Jilin University, Changchun, 130033 China; 8 Department of gastroenterology and hepatology, Hangzhou Red Cross Hospital, Hangzhou, 310003 China

**Keywords:** CRC, CLM, Proteogenomics, SAAV, Prognosis

## Abstract

**Background:**

Proteogenomic characterization and integrative and comparative genomic analysis provide a functional context to annotate genomic abnormalities with prognostic value.

**Methods:**

Here, we analyzed the proteomes and performed whole exome and transcriptome sequencing and single nucleotide polymorphism array profiling for 2 sets of triplet samples comprised of normal colorectal tissue, primary CRC tissue, and synchronous matched liver metastatic tissue.

**Results:**

We identified 112 CNV-mRNA-protein correlated molecules, including up-regulated COL1A2 and BGN associated with prognosis, and four strongest hot spots (chromosomes X, 7, 16 and 1) driving global mRNA abundance variation in CRC liver metastasis. Two sites (DMRTB1^R202H^ and PARP4^V458I^) were revealed to frequent mutate only in the liver metastatic cohort and displayed dysregulated protein abundance. Moreover, we confirmed that the mutated peptide number has potential prognosis value and somatic variants displayed increased protein abundance, including high MYH9 and CCT6A expression, with clinical significance.

**Conclusions:**

Our proteogenomic characterization and integrative and comparative genomic analysis provides a new paradigm for understanding human colon and rectal cancer liver metastasis.

**Trial registration:**

ClinicalTrials, NCT02917707. Registered 28 September 2016, https://clinicaltrials.gov/ct2/show/NCT02917707.

## Background

Colorectal cancer (CRC) is a significant contributor of cancer morbidity and mortality [1]. Almost half of CRC patients die within 5 years of diagnosis due to the development of recurrent disease and metastasis [2]. Therefore, it is important to illuminate the molecular basis of CRC liver metastasis (CLM) in hopes of developing new effective treatment modalities.

The Cancer Genome Atlas (TCGA) has characterized the genomic features of many types of human cancers, including CRC [3–5] and The Clinical Proteomic Tumor Analysis Consortium has also performed CRC-integrated proteomic analyses [6]. However, the primary genetic basis of CLM has not been fully elucidated. Understanding the genetic and proteogenomic differences between primary colon cancer and associated metastases to the liver is essential for discovering metastasis-specific molecular biomarkers and for devising a better therapeutic approach for this disease.

In the present work, we report a comprehensive molecular characterization of human CLM. Multi-platform integration revealed that CRC metastatic to the liver is driven by diverse alterations affecting multiple genes and pathways. Proteogenomic characterization and integrative and comparative genomic analysis provides a functional context to annotate genomic abnormalities with prognostic value, as well as a new paradigm for understanding human colon and rectal cancer liver metastasis.

## Methods

### Patient specimen acquisition

The study was examined and approved by the Ethics Committee of the Shanghai Tenth People’s Hospital, Tongji University School of Medicine (SHSY-IEC-PAP-16-24). This study was registered with ClinicalTrials.gov, number NCT02917707. Each participant provided their written informed consent to participate in this study. The inclusion criteria included: age ≤ 75 years with histologically proven CRC, no severe major organ dysfunction, WHO performance status of 0 or 1, or no prior cancer chemotherapy. The exclusion criteria included: age ≥ 76, severe major organ dysfunction, World Health Organization (WHO) performance status of > 1, or prior cancer chemotherapy. The morphology of primary CRC and paracarcinoma normal colorectal tissues was confirmed by two independent pathologists using cryostat frozen sections stained with hematoxylin and eosin. Follow-up data and statistics were recorded for all patients through Dec. 31, 2017.

### DNA and RNA extraction

Using a co-isolation protocol, DNA and RNA were purified simultaneously using the QIAGEN All Prep DNA/RNA Micro Kit (Qiagen, CA, USA) according to the manufacturer’s instructions. The nucleic acid concentration was determined using a Nanodrop1000 spectrophotometer (Thermo Fisher Scientific; Waltham, MA, USA), and the RNA purity was verified using 1.5% denaturing agarose gels.

### Protein extraction and analysis by LC-MS/MS

Fresh CRC tissues and para-tumor normal colorectal tissues (PN) were used for proteogenomic analysis. Three different parts of the same lesions for every sample were compared for data analysis and measurement of the variation caused by random biological effects. The samples were cut into small pieces (about 1 mm^3^) and rinsed in PBS to remove the blood. Then the tissues were homogenized in 4% SDS and 0.1 M DTT in 0.1 M Tris-HCl, pH 7.6 on ice, sonicated 10 times (80 w; 10 s sonication/5 s suspension), incubated for 3 min at 95 °C, and briefly sonicated. The protein concentrations of clarified lysates were determined using a fluorescence assay and then 200 μg of clarified lysates were proteolyzed on a 10 kDa filter (PALL Life Sciences, Shanghai, China) using the filter-aided sample preparation method [7]. The peptide samples were then desalted onto a solid-phase extraction cartridge. The lyophilized peptide mixture was re-suspended in water with 0.1% formic acid (*v*/v), and its content was estimated by ultraviolet light spectral density at 280 nm [8]. Then, 3 μg of the digest sample was analyzed by nano-liquid chromatography-tandem mass spectrometry on a LTQ Orbitrap Velos Pro mass spectrometer as previously described [9].

The acquired data from mass spectrometry runs were combined and searched against the UniProt Human database (05/2016, 153,652 entrys) using Maxquant software (version 1.3.0.5; http://maxquant.org/) as described [10]. Proteins were identified using the Andromeda peptide search engine integrated into the Maxquant platform. Trypsin-digested fragments were analyzed, allowing for a maximum of 2 missed cleavages. Carbamidomethyl cysteine was set as a fixed modification, with protein N-acetylation and methionine oxidation as variable modifications. Precursor ion tolerances were 20 ppm for first search and 6 ppm for a second search. The MS/MS peaks were de-isotoped and searched using a 20 ppm mass tolerance. The required minimum peptide length for identification was 7 amino acids, and the false discovery rate at the protein level, peptide level and site were set to 0.01. The normalized spectral protein intensity (label-free quantification) values were calculated for each protein group.

The Maxquant peptide and protein quantification result files were imported into Perseus software (version 1.5.1.6) to identify the differentially expressed proteins. After importing the quantitative data from ProteinGroups.txt into Perseus, a filtering criterion is set to keep the identified proteins with the quantified values of all ten reporter ions (no missing value) in the final identification list. The protein intensities are log_2_-transformed and normalized by subtracting the median intensity in each column/sample. Principal component analysis is performed based on protein intensities to differentiate groups. Two-samples tests coupled with Benjamini–Hochberg (FDR cutoff of 0.05) correction are performed to identify the differentially expressed proteins.

### RNA sequencing analysis

Six specimens from 2 CRC patients with metastasis (comprised of triplet sets of PN, primary CRC tumor samples with liver metastasis (MT), and synchronously matched liver metastasis focus tissues (LM)) and 3 specimens from 3 CRC patients without liver metastasis (NM) were obtained for RNA sequencing analysis. The mRNA libraries were separately generated from total RNA and constructed according to the standard Illumina RNA library preparation protocol (Illumina Inc., USA). Sequencing was performed on the Illumina Nextseq 500 platform according to the manufacturer’s instructions. Images generated by Nextseq 500 were converted into nucleotide sequences using a base call pipeline and stored in FASTQ format, and the raw reads were filtered prior to analyzing the data. Clean reads were mapped to reference *Homo sapiens* transcriptome sequences from the UCSC website (hg19) using Bowtie2 and Tophat 2.0.1 software. To annotate gene expression, reads per kilobases per million read values of each gene were calculated, and differentially expressed genes were extracted using this value. The formula for calculating these values was defined as: reads per kilobases per million read values = total exon reads / (mapped reads [millions] × exon length [kbp]).

### Chromosome microarray analysis and whole exome sequencing

Six specimens from 2 patients (including 2 triplet sets of primary MT, matched CLM and PN) were used for chromosome microarray analysis and whole exome sequencing analysis. DNAs and cRNAs were hybridized to the Affymetrix CytoScan HD Array as described and recurrent genomic regions with DNA copy gain and loss were identified using GISTIC, version 2.0 [11]. Genomic DNA was enriched for exonic regions using the SureSelect Biotinylated RNA Library. The sequences of captured libraries were generated as 90-bp pair-end reads on an Illumina Hiseq2000. Sequencing reads were processed and mapped to the reference GRCh37/hg19 human genome assembly using the Burrows-Wheeler Aligner as described [12]. Further processing, including duplicate removal, local realignment, base quality recalibration, and filtering, as well as the identification of SNVs and indels, was performed using the Genome Analysis Toolkit [13], SAM [14], and Picard tools (http://picard.sourceforge.net). Then, filters were applied to obtain higher confidence, and annotation and classification were performed using ANNOVAR [15]. The variant collection was excluded from positions reported in the 1000 Genomes Project and dbSNP. The mean sequencing depth in the target regions was 80.28× (range 71.5 to 92.85).

### Validation of point mutations by PCR and sanger sequencing

The reliability of the exome analysis and somatic variant identification strategies was assessed using PCR and Sanger sequencing. PCR was performed using the GeneAmp PCR System 9700 (Applied Biosystems, Foster City, CA, USA). About 20 ng template DNA from each sample was used per reaction. The products were sequenced, and all sequences were analyzed with the Sequencing Analysis Software Version 5.2 (Applied Biosystems).

### Assay design, PCR amplification and genotyping

A panel comprising 120 positive sites identified by Sanger sequencing were selected. These single nucleotide polymorphisms were located within genes of different functional categories. For the PCR amplification and single base extension reaction, primer pairs and extension primers were designed using Assay design suite v2.0. These primers were multiplexed and genotyped using the Sequenom MassARRAY platform integrating the iPLEXSBE reaction and MassARRAY technology (Agena Bioscience, San Diego, CA, USA) based on the MALDI-TOF MS assay [16].

### Hierarchical clustering, gene ontology (GO) and Kyoto encyclopedia of genes and genomes (KEGG) pathway analysis

Hierarchical clustering was performed using MEV software (http://mev.tm4.org/, v4.7.0, TIGR). The matrix was presented graphically by colouring each expression result on the basis of measured colour range: lower limit ‘0.0’ was coloured green, upper limit ‘369.5’ was coloured red and midpoint value ‘37.5’ was coloured black. Pearson correlation was used as distance metric and the complete linkage method was used. To identify genes/proteins that are specifically dysregulated in CLM, we fixed the cutoff at 2-fold with a *P* value less than 0.05. Dysregulated genes/proteins were subjected to GO analysis and KEGG pathway analysis by DAVID (http://david.ncifcrf.gov). Pathway analysis is used to map genes to KEGG pathways. The *P* value denotes the significance of the pathway correlations (*P* value < 0.05 is recommended).

### TCGA data acquisition and processing

We downloaded RNA-sequencing data from 379 CRC patients from TCGA portal (https://cancergenome.nih.gov/), 12 of which had liver metastasis at the time of diagnosis or during the five-year follow-up period, and 367 of which had CRC without metastasis to the liver. The mRNA expression levels were investigated in 379 CRC tissues and 32 PN tissues in TCGA datasets by Illumina HiSeq 2000 RNA Sequencing Version 2 analysis and normalized by the RSEM algorithm. Whole-exome sequencing mutation datasets were downloaded from TCGA data set to create a customized CRC mutation database. The clinical information recorded, including the patient’s characteristics, tumor characteristics, and overall and progression-free survival was assessed.

### Cell lines and transfection

Human CRC cell line SW480 were purchased from the Cell Bank of the Chinese Academy of Sciences (Shanghai, China) and cultured in DMEM media (Invitrogen, Carlsbad, USA) and supplemented with 10% (*v*/v) fetal bovine serum, 100 U/ml penicillin, and 100 mg/ml streptomycin. SW480 cell lines were routinely tested for mycoplasma contamination, and have been authenticated with short-tandem repeat analysis. Cell culture was conducted at 37 °C in a humidified 5% CO_2_ incubator. For *COL1A2* and *BGN* over-expression, the human full length cDNA with or without point mutation were cloned into the pMSCV-hygro vector. The SW480 cells with stable over-expression were polyclonal derivatives with hygromycin selection to avoid clonal variations in functional assays.

### Scratch-wound assay

The human CRC SW480 cells were conducted at 37 °C in a humidified 5% CO_2_ incubator and cells were grown into confluency in 6-well plates. The monolayer was artificially injured by scratching across the plate with a 200 ul pipette tip. The wells were washed 3 times to remove detached cells or cell debris. After 12 h, digital images were captured using a camera-equipped, inverted microscope (Carl Zeiss, Inc., Thorwood, NY, USA) and wound width measurements were subtracted from wound width at time zero to obtain the net wound closure.

### In vitro invasion assays

Corning Costar Transwell 24-well plates with 8-um-pore-size polycarbonate membrane filters (Costar, Cambridge, MA) coated with BD Matrigel matrix (Becton Dickinson, Bedford, MA) were maintained for 1 h at 37 °C, followed by the addition of 1 × 10^5^ transfected cells suspended in 200 μl medium with 1% serum into the top of each well insert. Normal growth medium was added to the bottom wells. The cells were allowed to migrate for 24 h at 37 °C. The migrated cells were fixed with 10% methanol for 15 min. The invading cells on the lower surface of the membrane were stained with 0.5% crystal violet for 5 min at room temperature. Random fields were photographed and the stained cells were counted under a microscope (Nikon Corporation).

### Statistical analysis

Data were expressed as means ± standard deviations. Categorical data were reported as numbers and percentages. F tests were used to assess the equality of variances for comparable groups. Paired t test, One-way analysis of variance (ANOVA), Kruskal-Wallis test, and χ^2^ tests were used to analyze mRNA expression. Forty four paired fresh CRC and PN tissues were used for survival analysis (Table 1). OS was measured from the date the patient underwent surgery until the date of death resulting from any cause or last known follow-up for patients still alive. DFS analysis was measured from the date the patient underwent surgery to the date of disease recurrence, death from any cause (ie, noncancer deaths were not censored), or until last contact with the patient. For time-to-event analyses, survival estimates were calculated by the Kaplan-Meier analysis, and groups were compared with the log-rank test. Clinical variables that were considered for single variable analyses were previously identified as confounding variables with impact on the prognosis of patients with colorectal cancer: age at diagnosis (continuous), sex, primary site (colon vs. rectum), pathological differentiation (well to moderate vs. poor), completeness of colorectal resection (R0 vs. R1), tumor size (≥ 5 cm vs. < 5 cm), number of primary foci (multiple vs. single) and necrosis (yes vs. no). The Spearman’s correlation coefficient was used to test the relationship of two independent groups. To identify genes/proteins that are specifically dysregulated in CLM, we fixed the cutoff at 2-fold with a *P* value less than 0.05. All calculations were performed with SPSS 20.0 software (SPSS Inc., Chicago, IL, USA).

**Table 1:** Summary of colorectal cancer patients demographic and clinical characteristics (*N* = 44)

Factor	Variables	Non-metastatic (*N* = 21)	Metastatic to liver (*N* = 23)
		Number (%)	Number (%)
Age	≥ 60	14 (66.7%)	11 (47.8%)
	< 60	7 (33.3%)	12 (52.2%)
Gender	Male	11 (52.4%)	10 (43.5%)
	Female	10 (47.6%)	13 (56.5%)
Primary site	Colon	9 (42.9%)	14 (60.9%)
	Rectum	12 (57.1%)	9 (39.1%)
Differentiation	Well	0 (0.0%)	0 (0.0%)
	Moderately	16 (76.2%)	16 (69.6%)
	Poorly	5 (23.8%)	7 (30.4%)
Completeness of colorectal resection	R0	21 (100.0%)	23 (100.0%)
	R1	0 (0.0%)	0 (0.0%)
Diameter	≥ 5 cm	10 (47.6%)	10 (43.5%)
	< 5 cm	11 (52.4%)	13 (56.5%)
Number of foci	Multiple	8 (38.1%)	9 (39.1%)
	Single	13 (61.9%)	14 (60.9%)
TNM stage	I-II	5 (23.8%)	5 (21.7%)
	III-IV	16 (76.2%)	18 (78.3%)
Necrosis	Yes	11 (52.4%)	10 (43.5%)
	No	10 (47.6%)	13 (56.5%)

## Results

### Identification of peptides and proteins associated with CLM

**Fig. 1:**
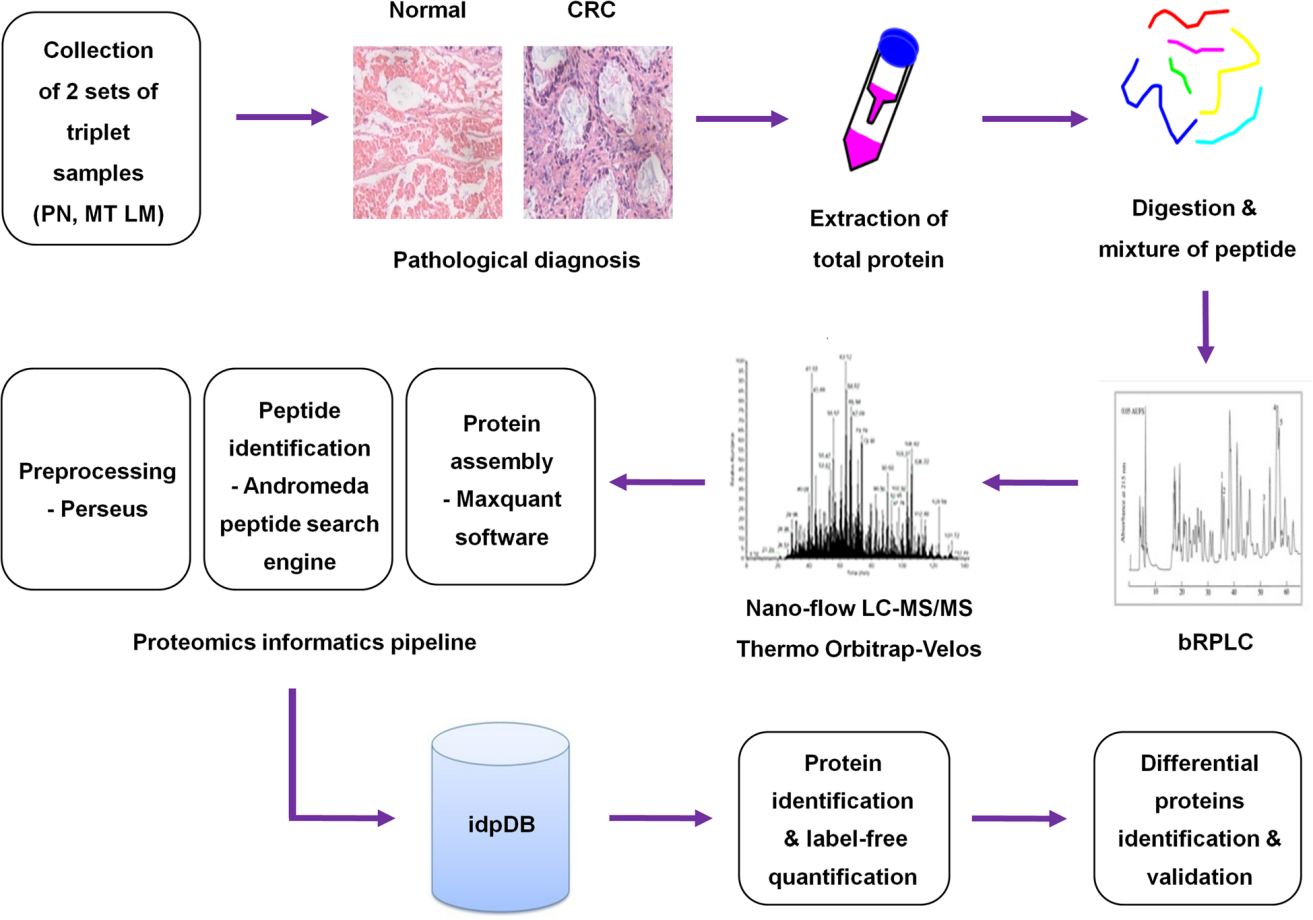
Mass-spectrometry-based proteomics workflow. Protein was extracted from fresh CRC and paired PN tissues and was used to generate tryptic digests. The resulting tryptic peptides were fractionated using off-line bRPLC Collected fractions were pooled and used with a Thermo Orbitrap-Velos MS instrument. Raw data were processed by Perseus software and then used for database and spectral library evaluation using the Andromeda peptide search engine. Identified peptides were assembled using Maxquant software. bRPLC, basic reverse-phase (high-pressure) liquid chromatography; CRC, colorectal cancer; PN paracarcinoma normal tissue

**Fig. 2:**
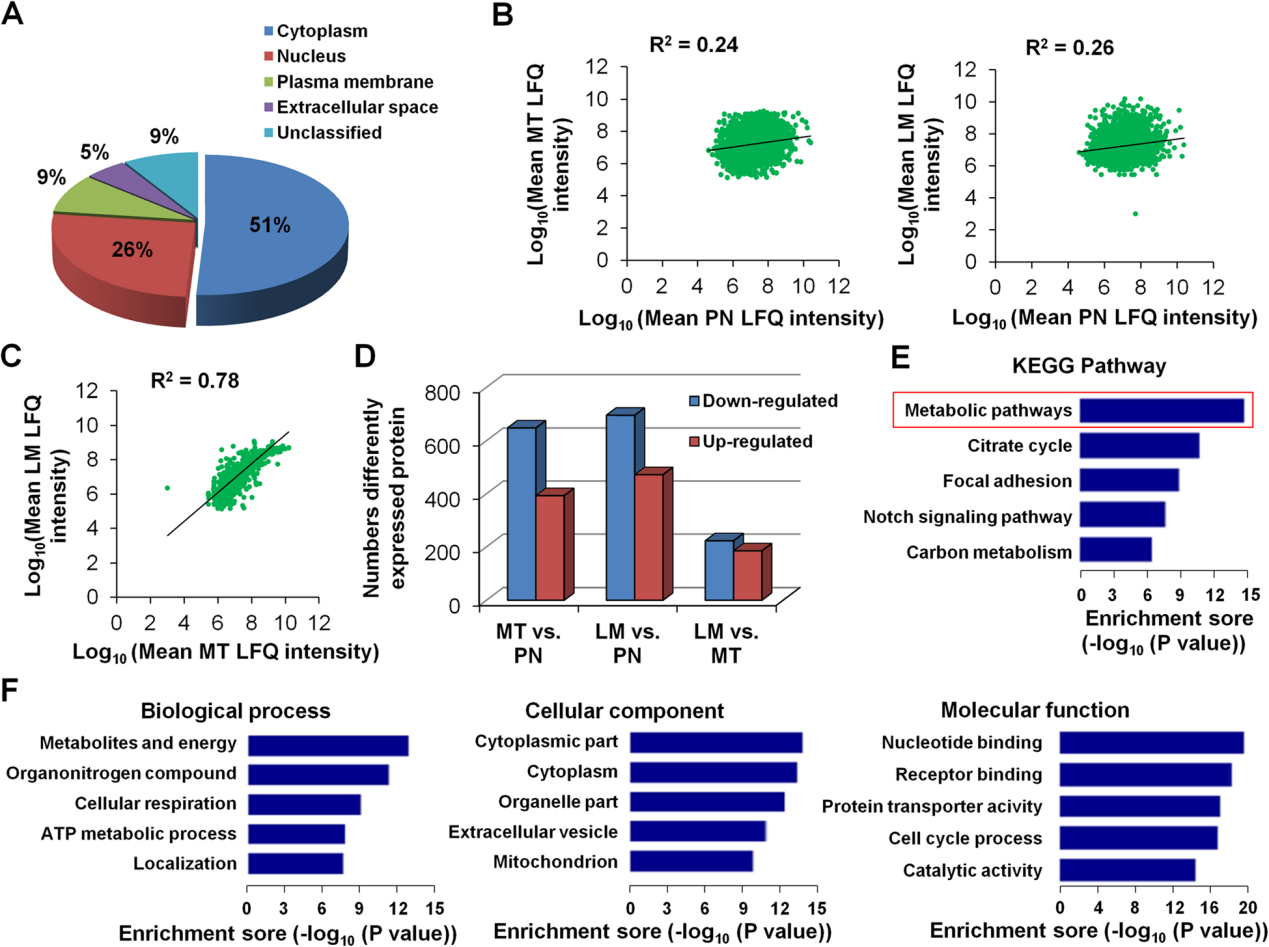
Differentially expressed proteins identified from 2 triplet sets of PN, MT and LM tissues. **a** The cellular distribution is shown for 4198 proteins identified by mass spectrometry, with a false discovery rate of 1.0%. Correlation analysis of the expression of 4198 proteins from MT/LM vs. PN (**b**) and LM vs. MT (**c**) samples from mass-spectrometry analysis. **d** Numbers of differently expressed proteins (≥2-fold difference; *P* value ≤0.05). **e** KEGG pathway analysis of the 1386 differentially expressed proteins. **f** Gene Ontology using STRING online analysis software classified the 1386 differentially expressed proteins in CLM according to their biological process, cellular components and molecular functions

Genomic features and proteomic analyses of CRC have been characterized; however, the primary genetic basis of CLM has not been fully elucidated, which is essential for discovering metastasis-specific molecular biomarkers and for devising a better therapeutic approach for this disease. To address these issues, we performed a nano-liquid chromatography-tandem mass spectrometry (LC-MS/MS)-based shotgun proteomics profiling of 2 sets of triplet samples comprised of para-tumor normal colorectal tissue (PN), primary CRC tissue (MT), and synchronous matched liver metastatic tissue (LM) (Fig. 1). Three different parts of the same lesions for every sample were compared for data analysis and measurement of the variation caused by random biological effects. A total of 596,234 spectra were used in the Andromeda engine search, and 26,375 unique peptides were identified in an assembly of 4198 protein groups with a protein-level false discovery rate of 1.0%. Ingenuity pathway analysis with all 4198 identified proteins showed that about 51% of the proteins were from the cytoplasm, 26% were from the nucleus, 9% were from the plasma membrane and 5% were from the extracellular space, whereas 9% of proteins remained unclassified (Fig. 2a). The random predicted cellular distribution of the proteins supports the quality of the sample preparation.

A scatter plot of protein abundance (label-free quantification intensity) between CRC and PN tissues showed that there was a great variation between the MT or LM tumors and PN tissue (Fig. 2b and c). However, the protein expression between the MT and LM group was positively correlated (R^2^ = 0.78) (Fig. 2d). These results suggest that liver metastasis focus and primary focus share similar protein profiles and that there are common molecular alterations at each stage of tumor development.

### Identification of significantly dysregulated proteins in CLM

To identify proteins that are specifically dysregulated in CLM, we fixed the cutoff at 2-fold with a *P* value less than 0.05. Among the 4198 proteins, a total of 1041 proteins were significantly altered between MT and PN tissue, 636 (61.09%) of which were down-regulated and 405 (38.91%) of which were up-regulated (Fig. 2a, left bars). There were 754 proteins with significantly difference in LM tissues when compared with PN tissues and 632 proteins with significantly difference in LM tissues when compared with MT tissues (Fig. 2d). Among that, 656 (47.33%) of which were down-regulated and 730 (52.67%) of which were up-regulated (Fig. 2a, middle and right bars) and 198 significantly differential expression proteins (DEproteins) (14.28%) involved in metabolic pathways (*P* = 3.28E-14) (Fig. 2e). These results suggest that metabolism-related pathways may play important roles in the liver metastasis of CRC.

To explore the functions of proteins that are dysregulated in CLM, we used DAVID analysis software to classify the Gene Ontology of the 1386 significantly altered proteins in LM tissues according to their molecular functions and cellular components and ranked them according to their biological processes (Fig. 2f). The top-ranked biological function was metabolites and energy, organonitrogen compound, cellular respiration, ATP metabolic process, localization, which suggests that metabolism-related biological function is associated with CLM (Fig. 2b).

### Identification of significantly dysregulated mRNAs in CLM

**Fig. 3:**
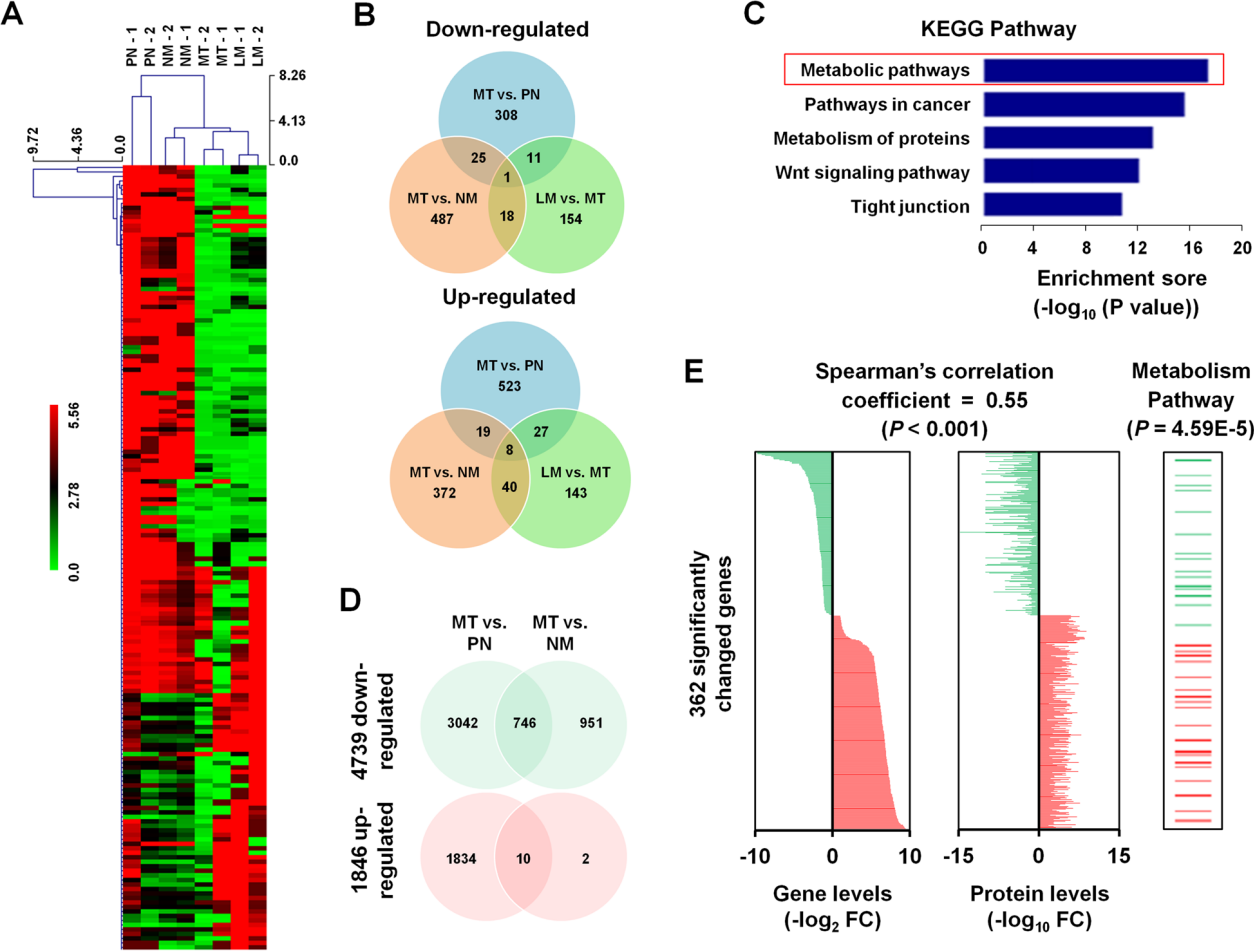
RNA sequencing and mRNA-protein correlation analysis. **a** Hierarchical clustering for RNA-sequencing data of 8 samples. **b** Significantly changed genes among three groups (MT vs. PN, MT vs. NM, and LM vs. MT) from RNA-sequencing data of 8 samples. **c** KEGG pathway classification enrichment analysis of 2136 differentially expressed genes in CLM. **d** Significantly changed genes among two groups (MT vs. PN and MT vs. NM) from TCGA RNA-sequencing data. **e** 362 significantly changed genes showed significant mRNA-protein correlation, with a mean Spearman’s correlation coefficient of 0.55. Among these, 48 genes were enriched in metabolism pathways

Next, we performed RNA sequencing to identify differentially expressed mRNAs (DEmRNAs) that are specifically dysregulated in CLM. Unsupervised hierarchical clustering of the expression data showed that the MT and LM tissues had closely related expression profiles when compared to para-tumor normal tissue or primary CRC tissue from CRC patients without liver metastasis (NM), suggesting clonal and genetic similarity for these pairs (Fig. 3a). We identified a total of 2136 genes significantly changed in LM (Fig. 3b) when compared to PN or NM groups, in which 462 genes (21.6%) were enriched in metabolism pathways (*P* = 1.58E-17) (Fig. 3c). Among them, 256 (55.41%) of which were down-regulated and 206 (44.59%) of which were up-regulated.

Moreover, analysis from TCGA sequencing dataset identified a total of 6585 significantly changed genes in CLM (Fig. 3d). A total of 5632 genes were significantly changed (3788 down-regulated and 1844 up-regulated) in the MT group compared to the PN group; and 1709 genes were significantly changed (1697down-regulated and 12 up-regulated) in the MT group compared to the CRC tumor samples without liver metastasis (NM) group. Among that, 1254 genes were in common with DEmRNAs identified in our study (58.7%).

### mRNA versus protein abundance in CLM

When compared with the 1386 DEproteins identified by LC-MS/MS, 362 DEmRNAs showed significant positive mRNA-protein correlation (Fig. 3e, left and middle panels). To determine whether the concordance between protein and mRNA variation is related to the biological function of the gene product, we performed KEGG enrichment analysis, which indicated that among the 362 significantly deregulated genes/proteins, 48 are enriched in metabolic pathways (*P* = 4.59E-5) (Fig. 3e, right panel). These findings further verify the role of metabolic pathway genes in CLM.

### Impact of copy number alterations in CLM

**Fig. 4:**
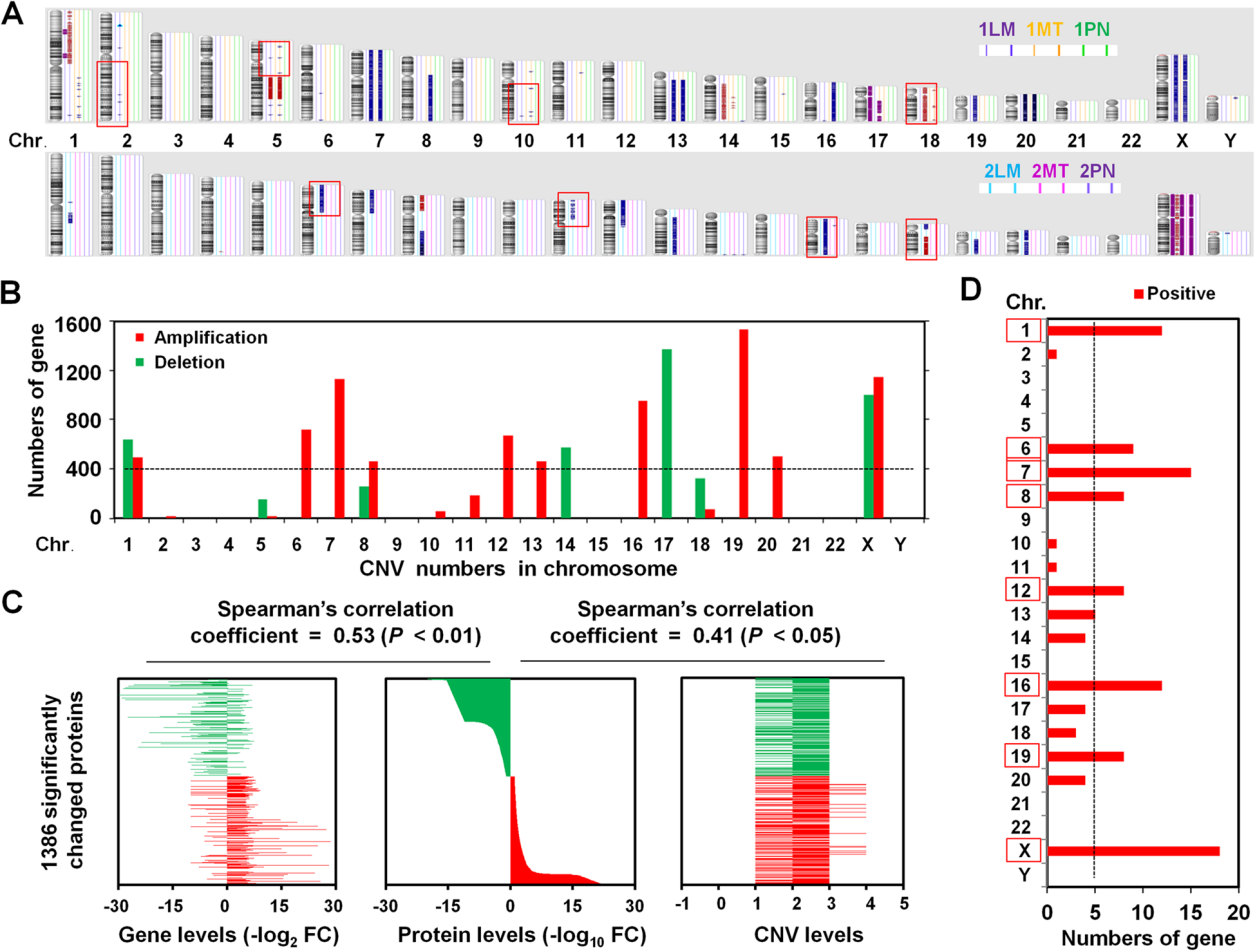
Effects of copy number alterations on mRNA and protein abundance. **a** Copy number alterations in 6 specimens from 2 patients, including 2 sets of primary MT, matched LM and PN, were identified by chromosome microarray analysis. Blue represents amplification, red represents deletion, and purple represents loss of heterozygosity. Red boxes indicate chromosomes that contain hot spots driving global mRNA variation abundance. **b** Chromosomal location of 321 regions (including 8424 genes) with significant focal amplification and 209 regions (including 2560 genes) with significant focal deletion in the LM or MT groups compared with the PN group. **c** Correlation analysis of the CNV-mRNA-protein abundance. The 1386 significantly changed proteins showed significant mRNA-protein correlation (multiple-test adjusted *P* < 0.01), with a mean Spearman’s correlation coefficient of 0.53 and CNV-protein correlation (multiple-test adjusted *P* < 0.05), with a mean Spearman’s correlation coefficient of 0.41. **d** Chromosomal location of 112 copy-number changed genes with positive CNV, mRNA and protein correlation

We further performed global copy number variation (CNV) analysis to identify likely gene targets of focal alterations and to explore the impact of CNVs on mRNA and protein abundance and the potential correlation with LM. PN samples displayed scarcely any gains or losses, however, relative to the PN group, the LM and MT groups had 321 regions of significant focal amplification and 209 regions of significant focal deletion (Fig. 4a, b). In addition to several previously well-defined arm-level changes associated with carcinogenesis of CRC^17^, gains of 2q, 5p, 6p, 10q, 11p and 16p/q and deleted 18p/q were identified to contain the mRNA abundance variation (Fig. 4a, b). When compared with the correlation of the protein-CNV correlation (Spearman’s correlation coefficient 0.41; *P* < 0.05), the correlation between protein level and mRNA expression was much stronger (Spearman’s correlation coefficient 0.53; *P* < 0.01) (Fig. 4c). These results suggest that the mRNA transcript abundance is a relatively reliable predictor of protein abundance differences, but that copy number alterations showed little consistency with the protein level.

To further examine the potential role of CNV, we calculated the number of genes/proteins that also had CNV alteration. When compared with the 362 significantly changed genes/proteins, 112 were found with changed copy-number (Fig. 4d). Among those, chromosomes 1, 6, 7, 8, 12, 16, 19 and X contained the strongly hot spots driving global mRNA abundance variation (Fig. 4d), which highlights the importance of these regions in CLM.

### Evaluation the prognostic and biological power of significantly dysregulated proteins in CLM

**Fig. 5:**
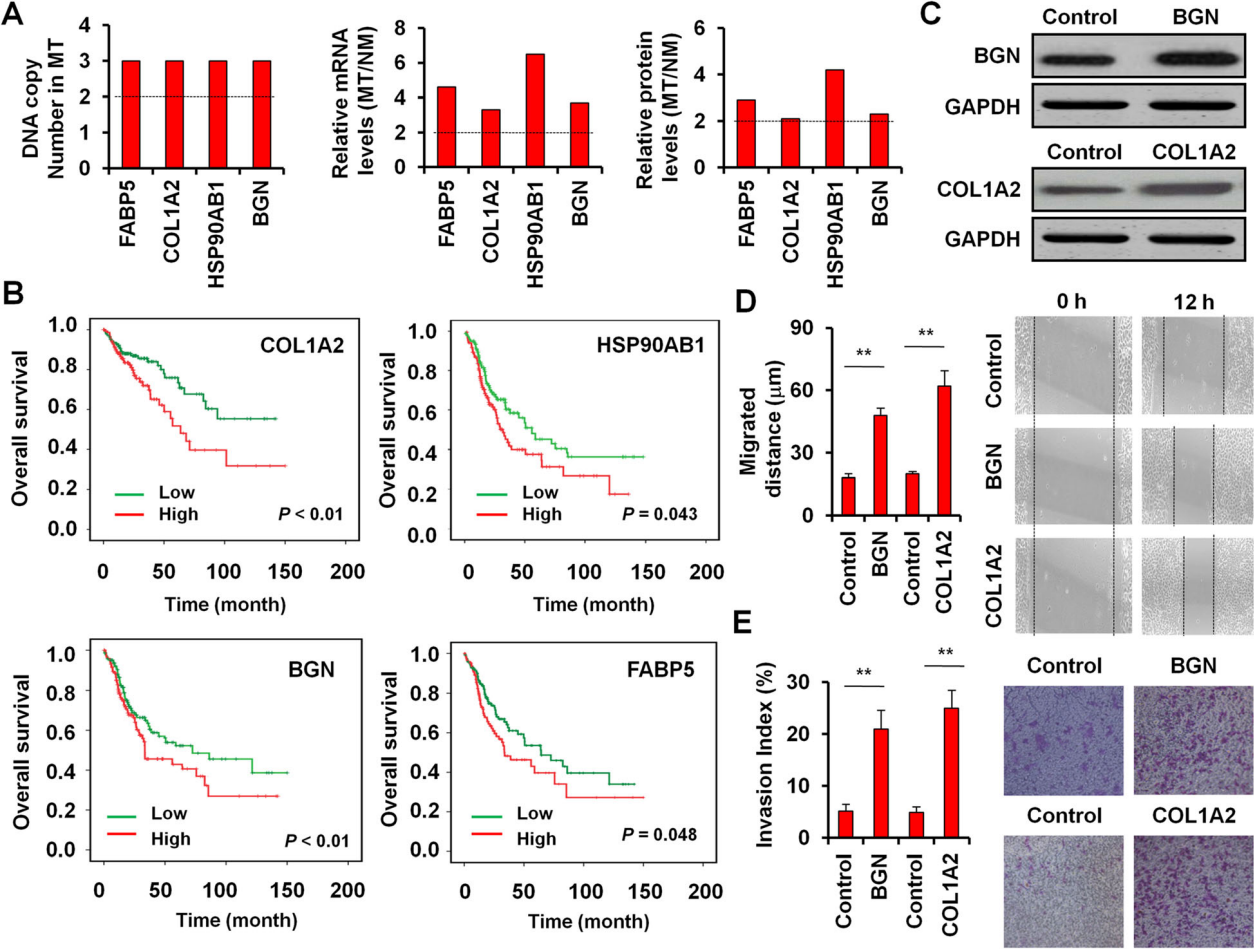
Two genes display significant focal amplification and increased mRNA-protein abundance with clinical significance and biological function. **a** Significant focal amplification and increased mRNA-protein abundance of 4 CNV-mRNA-protein correlated molecules in the MT group compared to the PN group. **b** The association of the expression levels of 4 CNV-mRNA-protein correlated molecules with overall and disease-free survival by Kaplan-Meier survival analysis. **c** Western blot analyze for COL1A2 or BGN overexpression in CRC cell line SW480. Wound-healing assay (**d**) and migration abilities (**e**) of the parental and COL1A2 or BGN overexpressed SW480 cells

We next evaluated the clinical significance of 286 CRC patients from TCGA database for the 112 CNV-mRNA-protein correlated molecules. Our results showed that 4 up-regulated genes (*HSP90AB1*, *COL1A2*, *FABP5* and *BGN*), which located in CNV hotspots (located in 6p21.1, 7q21.3, 8q21.13 and Xq28, respectively) were associated with prognosis of CRC patients (Fig. 5a). Kaplan-Meier survival analysis confirmed that high expression of *HSP90AB1*, *COL1A2*, *FABP5* or *BGN* was significantly associated with a shorter overall survival (*P* < 0.05) (Fig. 5b). Among those, high expression of *COL1A2* and *BGN* was extremely significantly associated with a shorter overall survival (*P* < 0.01) (Fig. 5b). Moreover, high expression of *COL1A2* or *BGN* was positively associated with disease-free survival (*P* < 0.05) as determined by Kaplan-Meier survival analysis.

To investigate the biological role of these CNV-mRNA-protein correlated genes, which associated with the prognosis in CRC progression and liver metastasis, we established the CRC cell line SW480 to stably overexpress COL1A2 or BGN to perform the gain-of-function studies in vitro (Fig. 5c). We then tested the effect of cell migration by COL1A2 or BGN overexpression via wound-healing assay and observed significant improvement of cell motility by COL1A2 or BGN (*P* < 0.01) (Fig. 5d). By two-chamber transwell assays, we also showed that forced expression of COL1A2 or BGN markedly enhanced the transwell invasiveness of SW480 (*P* < 0.01) (Fig. 5e).

### Somatic coding mutations in primary and metastatic CRC

**Fig. 6:**
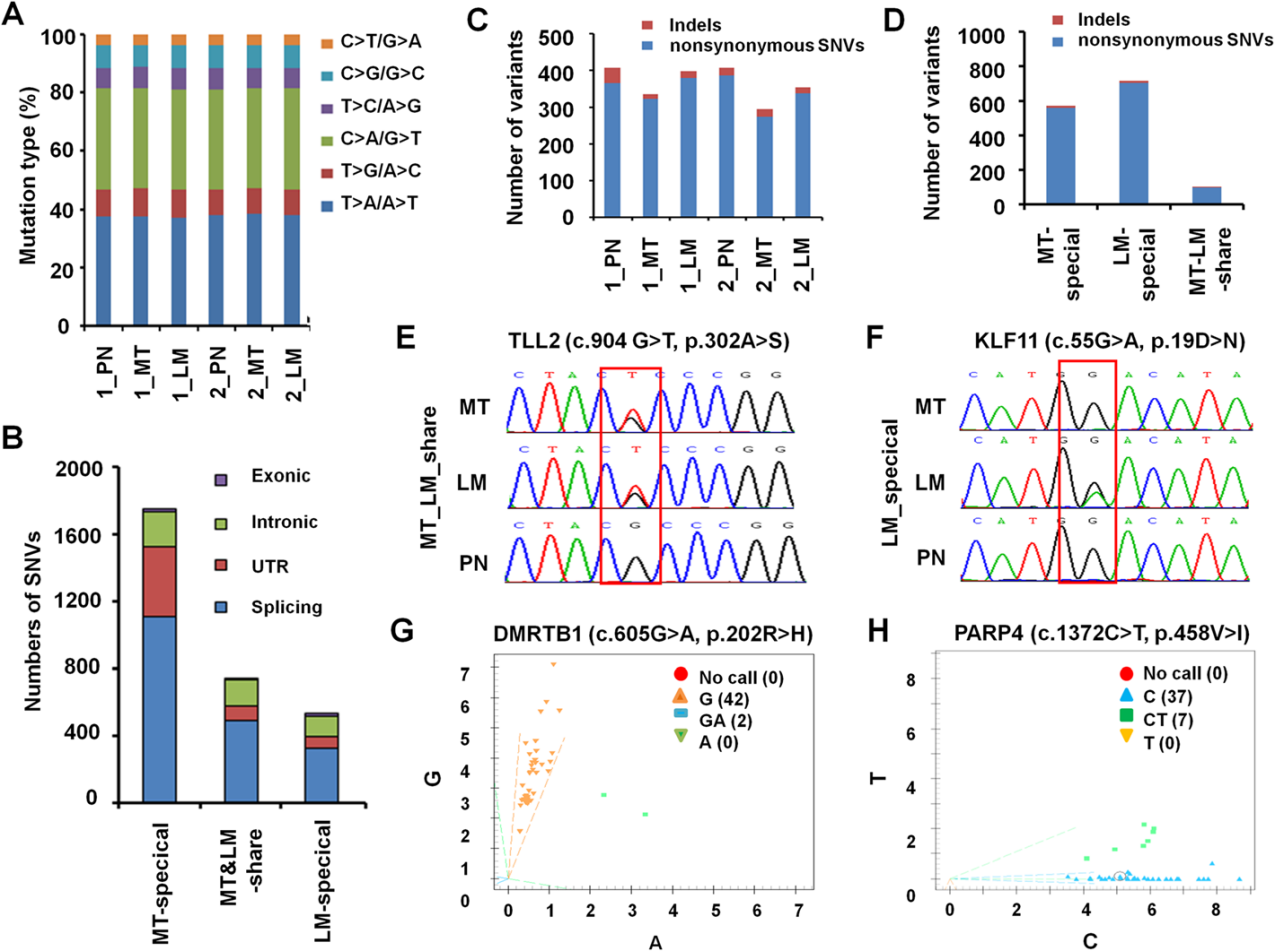
Somatic coding mutations in paired normal colorectal samples, metastatic CRC and hepatic metastatic focus. **a** Mutation types in 6 specimens from 2 patients, including 2 sets of PN, primary MT, and synchronous matched LM. T > A transversions were the most common nucleotide substitution. **b** Distribution of SNVs in exonic, intronic, UTR and splicing regions based on our RNA-sequencing data. **c** Distribution of indels and non-synonymous SNVs in 6 samples from 2 patients, including 2 sets of PN, primary MT, and synchronous matched LM specimens. **d** Numbers of MT-specific, LM-specific and MT & LM-shared indels and nonsynonymous SNVs. Mutations involved in CLM including the TLL2^A302S^ mutation, which was identified in both the MT and LM groups (**e**) and the KLF11^D19N^ mutation, which was specific for the LM cohort (**f**). Two frequently mutated sites (DMRTB1^R202H^ and PARP4^V458I^) in the MT cohorts were identified by SNP genotype analysis, with separate mutation rates of 5.26% (2/38) (**g**) and 17.5% (7/40) (**h**)

To provide a comprehensive understanding of genetic abnormalities occurring in CLM, we used massively parallel paired-end sequencing technology to perform whole-exome solution-based hybrid capture sequencing of 2 triplet sample sets. The mean sequencing depth in the target regions was 80.28× (range 71.5 to 92.85). Analysis of the whole-exome sequencing data identified 27,778.5 mean point mutations (range 26,323 to 29,126). There were a variety of types of mutations identified, with T > A transversion being the most common nucleotide substitution (Fig. 6a). The distribution of CLM-related SNVs is shown in Fig. 6b. After filter analysis and exclusion of synonymous mutations, the numbers of indels and non-synonymous SNVs were calculated (Fig. 6c). In addition to some previously reported mutations, such as those in TP53, APC, KRAS, and PIK3CA [5], we identified 97 MT and LM-shared point mutations and 701 point mutations only existed in MT (Fig. 6d).

We further assessed the somatic gene mutations in an extended validation group of 44 paired normal colorectal tissues and CRC tissues by Sanger sequencing and nucleotide polymorphism genotype analysis. Subsequently, 175 nonsynonymous mutations within 171 genes were further verified.

In addition to the expected APC, TP53, SMAD4, PIK3CA and KRAS mutations, we found some new mutations that have not been reported to be involved in CLM including the TLL2^A302S^ mutation, which was identified in both the MT and LM groups (Fig. 6e), and the KLF11^D19N^ mutation, which was specific for the LM cohort (Fig. 6f). Moreover, our single nucleotide polymorphism genotype analysis revealed that 2 sites (FABP5^A2T^ and HSP90AB1^E299N^) were frequent mutated only in the MT cohort, with mutation rates of 4.55% (2/44) (Fig. 6g) and 15.9% (7/44) (Fig. 6h), which suggest their potential roles in CLM.

### Single amino acid variants (SAAVs) in CRC

**Fig. 7:**
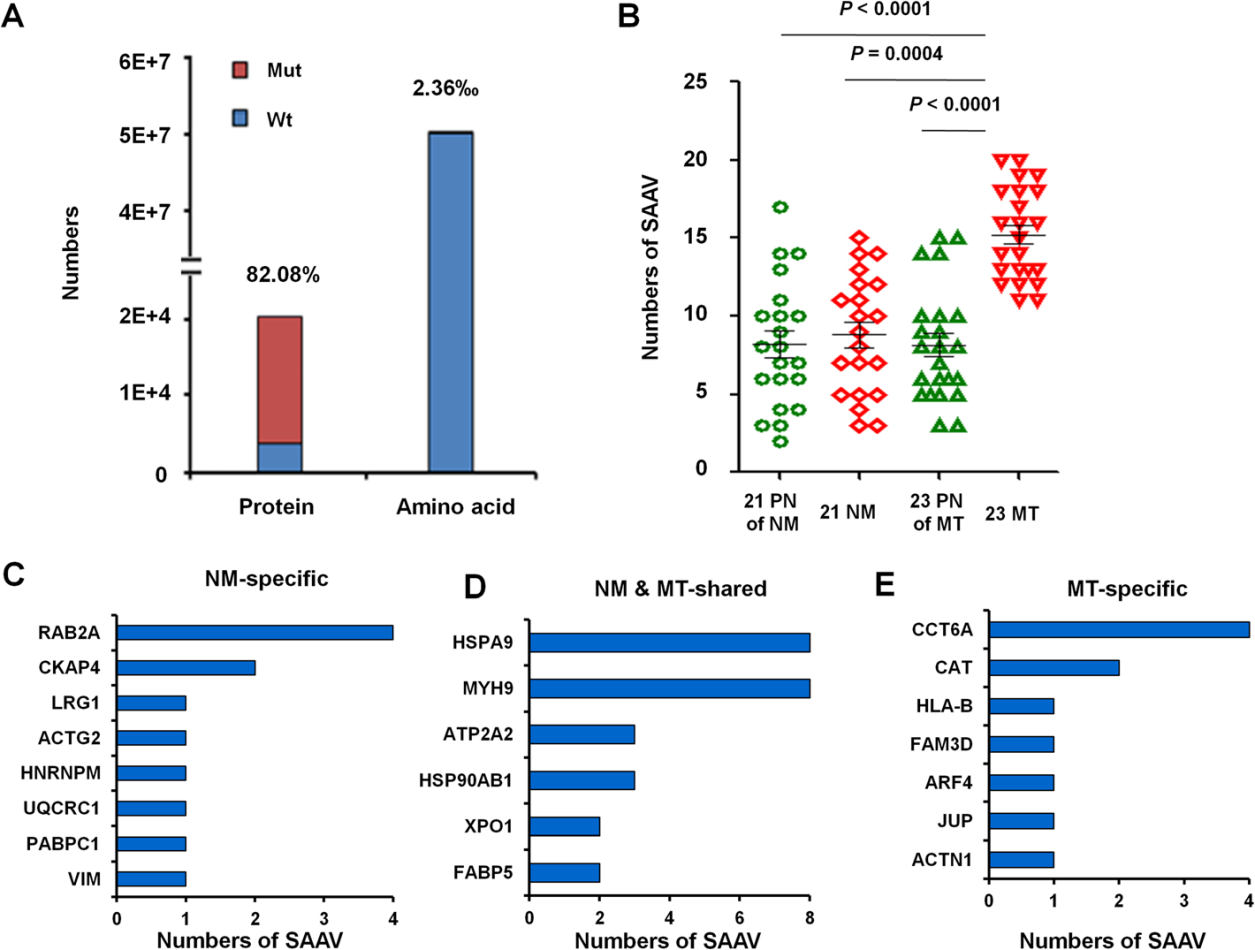
Numbers of SAAVs in paired PN, NM or MT samples. **a** The proportion of mutated proteins and amino acids in CRC samples were calculated by comparing LC-MS/MS data for the standard protein library and SAAV library. **b** Numbers of SAAVs in 21 NM, 23MT and their PN colorectal tissues. Numbers of NM-specific (**c**), MT-specific (**d**) and NM & MT-shared SAAVs (**e**).The mutated pepides were identified by comparing LC-MS/MS data for the standard protein library and SAAV library

A fundamental goal of proteogenomics is to identify protein coding alterations that are expressed at the protein level. However, standard database search approaches cannot identify variant peptides from MS/MS data. Therefore, we created a customized mutation database to search for SAAVs in CRC. A SAAV library was prepared using 113,844 mutated sites in CRC tissues from cBioport and our whole exome sequencing data, and 16,581 mutated proteins were identified, which constitute 82.08% of 20,201 proteins in the CRC standard protein library (Fig. 7a).

We determined the total numbers of mutated and non-mutated peptides and tumor-specific mutant peptides and found that mutated peptide numbers in MT samples were significantly increased (Fig. 7b), which indicates that the mutated peptide number has potential predictive value for CRC liver metastasis. Among those, 12 SAAVs in 8 proteins occurred only in NM patients (Fig. 7c; Table 2); and 13 proteins in 18 MT patients had 26 SAAVs; of which, 26 SAAVs in 6 proteins occurred in both NM and MT samples (Fig. 7d; Table 3), and 11 SAAVs in 5 proteins only occurred in MT samples (Fig. 7e; Table 4).

**Table 2:** SAAVs in NM-specifical sample

Protein name	Accession number	Wild Type peptide	Mutant peptide	Site of peptide	Site of SAAV	No. of SAAVs
RAB2A	P61019	IQEGVFDIDNEANGIK	IQEGVFDINNEANGIK	P61019_171_186	D179N	4
CKAP4	Q07065	ITIQAITEK	IAIQAITEK	Q07065_347_355	T348A	2
VIM	P08670	IIEEMIQR	IQEEMIQR	P08670_189_196	I190E	1
PABPC1	P11940	GFGFVCFSSPEDATK	GFGFVCFSSPEEATK	P11940_334_348	D345E	1
UQCRC1	P31930	ICTSVTESEVAR	ICTSATESEVAR	P31930_379_390	V383A	1
HNRNPM	P52272	INDIISNAIK	INEIISNAIK	P52272_372_381	D374E	1
ACTG2	P63267	CEEETTAPVCDNGSGICK	CEEETTAIVCDNGSGICK	P63267_2_19	P9I	1
LRG1	P02750	NAITGIPSGIFQASATIDTIVIK	NAITGIPPGIFQASATIDTIVIK	P02750_126_148	S133P	1

**Table 3:** SAAVs in NM & MT-share sample

Protein name	Accession number	Wild Type peptide	Mutant peptide	Site of peptide	Site of SAAV	No. of SAAVs
MYH9	P35579	AGVIAHIEEER	AGVITHIEEER	P35579_765_775	A769T	8
HSPA9	P38646	EQQIVIQSSGGISKDDIENMVK	EQQIVIQSSGGISNDDIENMVK	P38646_542_563	K555 N	8
HSP90AB1	P08238	NPDDITQEEYGEFYK	NPDDITQDEYGEFYK	P08238_292_306	E299N	3
ATP2A2	P16615	DIVPGDIVEIAVGDK	DIVPGDNVEIAVGDK	P16615_144_158	I150N	3
FABP5	Q01469	ATVQQIEGR	TTVQQIEGR	Q01469_2_10	A2T	2
XPO1	O14980	NVDIIKDPETVK	NVDIIQDPETVK	O14980_675_686	K680Q	2

**Table 4:** SAAVs in MT-specifical sample

Protein name	Accession number	Wild Type peptide	Mutant peptide	Site of peptide	Site of SAAV	No. of SAAVs
CCT6A	P40227	NAIDDGCVVPGAGAVEVAMAEAIIK	NAIDDGCVVPGAGAVEVAMAEAINK	P40227_400_424	I423N	4
CAT	P04040	NISVEDAAR	NISVEDVAR	P04040_244_252	A250V	2
ACTN1	P12814	VGWEQIITTIAR	VGWEQIITTITR	P12814_715_726	A725T	1
JUP	P14923	TMQNTSDIDTAR	TMQNTNDIDTAR	P14923_192_203	S197 N	1
ARF4	P18085	HYFQNTQGIIFVVDSNDR	HYFQNTQGIIFVVDSDDR	P18085_80_97	N95D	1
FAM3D	Q96BQ1	AFDMYSGDVMHIVK	SFDMYSGDVMHIVK	Q96BQ1_118_131	A118S	1
HLA-B	P01889	FISVGYVDDTQFVR	FIAVGYVDDTQFVR	P01889_46_59	S48A	1

**Fig. 8:**
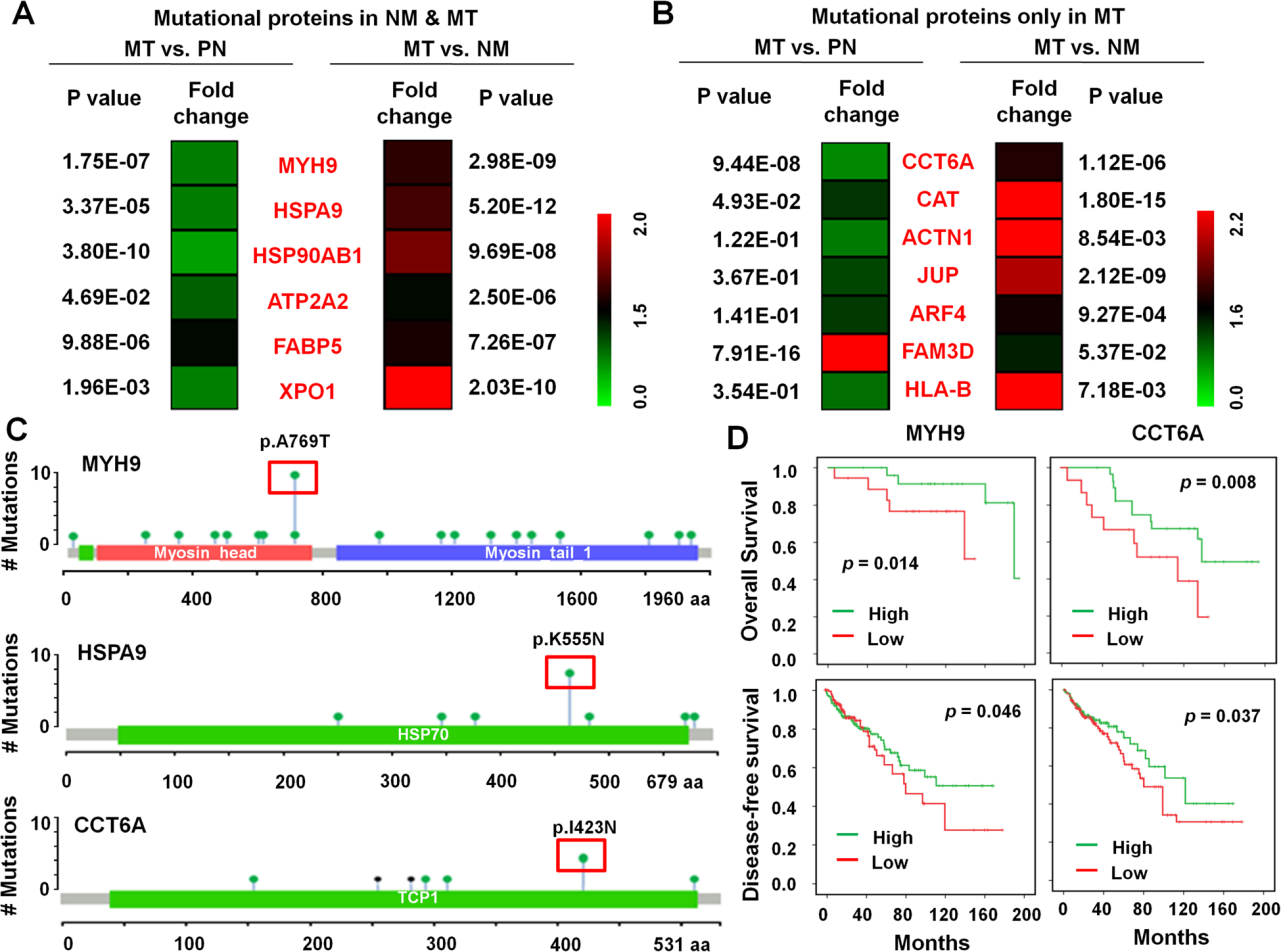
Impact of SNVs on protein abundance. **a** Expression levels of 6 NM & MT-shared mutated proteins (including MYH9, HSPA9, HSP90AB1, ATP2A2, FABP5 and XPO1) in MT samples compared with NM or PN samples. **b** Expression levels of 7 MT-specific mutated proteins (including CCT6A, CAT, ACTN1, JUP, ARF4, FAM3D and HLA-B) in MT samples compared with NM or PN samples. **c** Mutation sites of 3 genes contained within the strongest mutational hot spots. **d** Univariate survival analysis of overall survival and disease-free survival based on MYH9 and CCT6A levels

To further evaluate the potential role of SAAVs, we examined the expression levels of the proteins with SAAVs. The expression of 6 NM & MT-shared (Fig. 8a) and 8 MT-specific mutated proteins was upregulated in CRC. The sites of the most frequently mutated three proteins, MYH9^A769T^, HSPA9^K555N^ and CCT6A^I423N^, are shown (Fig. 8c). Furthermore, high MYH9 and CCT6A expression were each associated with shorter overall survival and disease-free survival (*P* < 0.05; Fig. 8d), which indicates that they have potential predictive values for CRC liver metastasis.

## Discussion

CRC is the third most common malignancy and the second leading cause of cancer deaths in many countries. It develops from a benign adenomatous polyp into an invasive cancer, and nearly 50% of CRC patients develop CLM [17]. Without treatment, patients with colorectal hepatic metastases have a median survival of only 5–10 months, with less than 0.5% surviving beyond 5 years [18].

The molecular pathogenesis of CRC is associated with a variety of genetic changes that lead to the aberrant activation of proto-oncogenes and inactivation of tumor suppressor genes [19]. Characterization of CRC genomes has been elaborated by large-scale next-generation sequencing, which has yielded important insights into the genes and mechanisms that contribute to cancer development and progression. A handful of recurrently mutated genes, including APC, KRAS, TP53, and SMAD4, have been discovered by this method [20]. According to the classical tumor progression model of sporadic CRC proposed by Fearon and Vogelstein, APC mutation is involved in adenoma formation, followed by KRAS oncogenic mutation that promotes the transition from intermediate adenomas to carcinomas, with TP53 inactivation as a late event [21]. Subsequently, mutations in individual genes (including SMAD4) facilitate CRC metastasis [22]. Leveraging the next generation sequencing technology, TCGA Network has reported the common occurrence of mutations in additional genes, such as ARID1A, SOX9 and FAM123B, which also demonstrate that CRC is a highly genetically heterogeneous disease at the population level [5].

Understanding the genetic differences between primary colon cancer and their metastases to the liver is essential for devising a better therapeutic approach for this disease [23]. Therefore, research efforts have shifted from identifying driving mutations of carcinogenesis to genetic abnormalities during CRC progression in order to provide valuable insights into the clonal relationship and genetic differences between primary CRCs and matched colorectal liver metastasis [24]. A recent study reported high genomic concordance between primary colorectal carcinoma and metastases, which indicate that somatic mutations may accumulate within the microenvironment of a primary cancer before disseminating to their metastatic sites [25]. Consistent with this hypothesis, in this study, we employed primary CRC tumor samples from patients with liver metastasis to trace progressive disease and combined CNV, mRNA and protein profiling data to identify potentially relevant genes in amplified chromosomal regions. Our results revealed the importance of chromosomes X, 7, 16 and 1, which contain the four strongest hot spots driving global mRNA abundance variation. These results also provided new insights into the potential roles of PFDN4 and COL1A2 in CLM. We also created a customized mutation database of CRC to identify SAAVs that occur during CRC metastases to the liver. The results indicate that the mutated peptide number has potential prognosis value, which can be broadly extended to understand roles of SAAVs in other cancers.

## Conclusions

To the best of our knowledge, this is the first comprehensive study to use proteogenomic profiling of primary CRCs from patients with or without liver metastasis to define the dominant events of metastatic lesions in terms of their expression and mutation. Our comprehensive integrative analysis of 44 colorectal tumor and normal pairs provides a number of insights into the biology of CLM and identifies potential therapeutic targets. Moreover, our characterization of the annotated metastatic CRC proteome clarifies the power of integrating genomics (SNVs) and proteomics (SAAVs). This approach provides new insights into the roles of these protein alterations in CLM, which can be broadly extended to understand the roles of protein mutation in other cancers.

## Abbreviations

CLM: Colorectal cancer liver metastasis
CNV: Copy number variation
CRC: Colorectal cancer
DEmRNAs: Differentially expressed mRNAs
DEproteins: Differential expression proteins
LC-MS/MS: Liquid chromatography-tandem mass spectrometry
LM: Synchronous matched liver metastatic tissue from CRC patients
MT: Primary CRC tumor tissue from CRC patients with liver metastasis
NM: Primary CRC tissue from CRC patients without liver metastasis
PN: Para-tumor normal colorectal tissue
SAAVs: Single amino acid variants
TCGA: The Cancer Genome Atlas
