# Correction to: Proteogenomic characterization and comprehensive integrative genomic analysis of human colorectal cancer liver metastasis

**DOI:** 10.1186/s12943-019-1005-3

**Published:** 2019-04-02

**Authors:** Yu-Shui Ma, Tao Huang, Xiao-Ming Zhong, Hong-Wei Zhang, Xian-Ling Cong, Hong Xu, Gai-Xia Lu, Fei Yu, Shao-Bo Xue, Zhong-Wei Lv, Da Fu

**Affiliations:** 1 Central Laboratory for Medical Research, Shanghai Tenth People’s Hospital, Tongji University School of Medicine, Middle 301 Yanchang Road, Shanghai, 200072 China; 2 Shanghai Engineering Research Center of Molecular Therapeutics and New Drug Development, College of Chemistry and Molecular Engineering, East China Normal University, Shanghai, 200062 China; 3 Department of Nuclear Medicine, Shanghai Tenth People’s Hospital, Tongji University School of Medicine, Shanghai, 200072 China; 4 Institute of Health Sciences, Shanghai Institutes for Biological Sciences, Chinese Academy of Sciences, Shanghai, 200025 China; 5 Department of Tumor Radiotherapy, Jiangxi Provincial Tumor Hospital, Nanchang, 330029 China; 6 Analytical Chemistry Platforms, Shanghai Institute efor Advanced Immunochemical Studies, ShanghaiTech University, Shanghai, 201210 China; 7 Tissue Bank, China-Japan Union Hospital, Jilin University, Changchun, 130033 China; 8 Department of gastroenterology and hepatology, Hangzhou Red Cross Hospital, Hangzhou, 310003 China

**Correction to: Mol Cancer (2018) 17:139**

**https://doi.org/ 10.1186/s12943-018-0890-1**

Following publication of the original article [1], the authors reported an error in affiliation 5. The updated address is shown in the affiliation section. The authors would like to apologize for this error.
